# Supplementary material for: Abiotic synthesis of graphitic carbons in the Eoarchean Saglek-Hebron metasedimentary rocks
Source: Nat Commun. 2024 Jul 6;15:5679. doi: 10.1038/s41467-024-50134-1 (PMC11227522; doi:10.1038/s41467-024-50134-1)
Supplement: Supplementary file 1 — Supplementary Information [file 41467_2024_50134_MOESM1_ESM.pdf]

Supplementary Information for

Abiotic synthesis of graphitic carbons in the Eoarchean Saglek-Hebron metasedimentary rocks

Zixiao Guo[1,2,*], Dominic Papineau[2,3,4,5], Jonathan O'Neil[6], Hanika Rizo[7], Zhong-Qiang Chen[5], Xincheng Qiu[5], and Zhenbing She[5]

[1] *School of Geographical Sciences, Hebei Key Laboratory of Environmental Change and Ecological Construction, Hebei Normal University, Shijiazhuang 050024, China*

[2] *Department of Earth Sciences, University College London, London, WC1E 6BT, UK*

[3] *London Centre for Nanotechnology, University College London WC1H 0AH UK*

[4] *Centre for Planetary Sciences, University College London, WC1E 6BT UK*

[5] *State Key Laboratory of Biogeology and Environmental Geology, China University of Geosciences (Wuhan), Wuhan 430074, PR China*

[6] *Department of Earth and Environmental Sciences, Ottawa-Carleton Geoscience Centre, University of Ottawa, Ottawa, ON, K1N 6N5, Canada*

[7] *Department of Earth Sciences, Ottawa-Carleton Geoscience Centre, Carleton University, Ottawa, ON, K1S 5B6, Canada*

** Corresponding author: zxguo@hebtu.edu.cn (Z. Guo)*

The file includes:

Supplementary Note 1: Geological setting

Supplementary Figures 1-8

Supplementary References

Supplementary Note 1: Geological setting

The Saglek-Hebron Complex (SHC), located in the west end of the North Atlantic Craton along the east coast of northern Labrador, Canada, is one of the rare geological terrains preserving Eoarchean rocks on Earth. It is a granite-greenstone terrane mostly composed of orthogneisses from the trondhjemite-tonalite-granodiorite (TTG) series, and including enclaves of volcano-sedimentary successions (Supplementary Fig. 1). Supracrustal rocks contain chemical sedimentary rocks including carbonate rocks, chert and banded iron formation (BIF), and clastic sedimentary rocks including quartzite, pelitic and psammitic rocks and conglomerate, as well as igneous mafic and ultramafic rocks[1-3]. The SHC has experienced a complex and protracted thermal history with discontinuous magmatic and metamorphic events[4-6]. To the west of the Handy fault (Supplementary Fig. 1), the SHC rocks have been metamorphosed to granulite facies, while the east includes amphibolite facies rocks[1], with the southeastern area believed to have reached granulite facies conditions before being retrogressed to amphibolite facies. Therefore, the SHC, similar to other Eoarchaean terrains, has been subjected to severe deformation and upper amphibolite to granulite facies metamorphism[6,7].

The supracrustal rocks are generally divided into two distinct assemblages of different ages, based on the presence or absence of crosscutting Paleoarchean dykes[2]. The oldest supracrustal package, called the Nulliak unit, yielded Sm-Nd and Lu-Hf isochron ages suggesting an Eoarchean age of nearly 3.8 Ga, while the younger Upernavik supracrustal unit is more consistent with an age of ~3.4 Ga[8,9]. Banded iron formations appear to only be found in the Nulliak assemblage[10]. The SHC orthogneisses can be divided into multiple units, which include the ~3.9 Ga Iqaluk gneiss, the ~3.75 Ga Uivak I and ~3.6 Ga Uivak II gneiss, the ~3.3 Ga Iluilik gneiss, the ~3.2 Ga Lister gneiss, and late ~2.7 Ga granitic intrusions[6]. The oldest U-Pb age on zircon that has been reported for the SHC rocks is from an orthogneiss dated at 3920 ± 49 Ma[11], but this age has been called into question by Whitehouse et al. (2019)[12] who argued that the U-Pb data presented by Shimojo et al. (2016)[11] is more consistent with an age of 3865 ± 4 Ma. Moreover, an age of 3869 ± 6 Ma was subsequently obtained on the same rocks from the same outcrop[6]. It is also unclear whether the age of this ~3.9 Ga orthogneiss places a solid geochronological constraint on the Saglek-Hebron BIF. Based on the presence of a small portion of mafic rock within this felsic gneiss, it has been suggested that the

SHC supracrustal rocks (and therefore the BIF) were older than 3.9 Ga[11], but this geological relationship has been the object of skepticism[12]. Nevertheless, Sm-Nd and Lu-Hf isotopic compositions, as well as U-Pb zircon dating suggest an Eoarchean age of at nearly 3.8 Ga for the Nulliak supracrustal assemblage[8,9,13], making these rocks among the oldest known on Earth.

Supplementary Figures

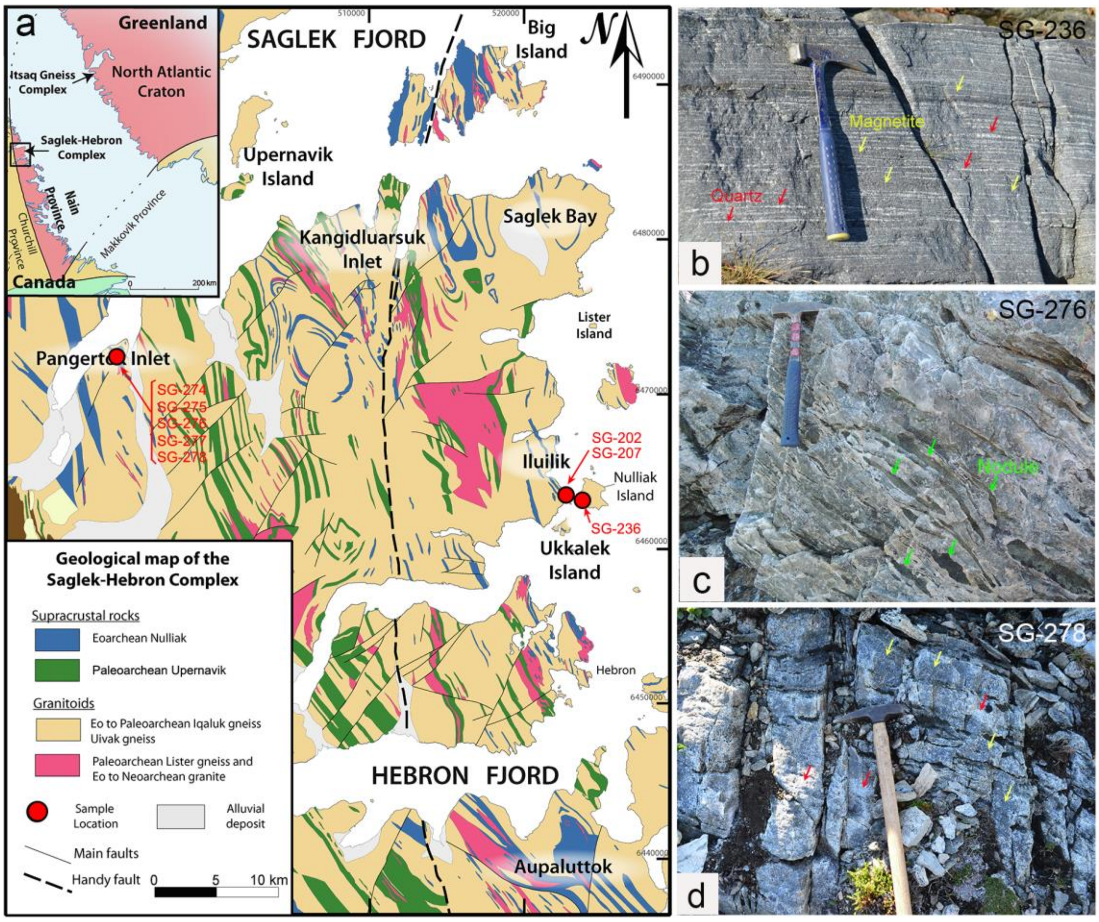

Supplementary Figure 1. Geological maps and sampling locations in the Saglek-Hebron Gneiss Complex (SHC). **a** Simplified geological map of the SHC showing the sampling localities in the study areas (red circles; modified after Wasilewski et al., 2021[6], with permission from Elsevier). Outcrop photos: **b** a strongly magnetic banded iron formation (BIF) showing finely laminated banding of quartz (red arrows) and magnetite (yellow arrows) (SG-236); **c** magnetite-bearing chert comprising deformed, amphibole-rich, lens-shaped nodules (green arrows) (SG-276); **d** quartz-magnetite-biotite BIF showing strongly magnetic unit with a larger proportion of magnetite in the darker areas (yellow arrows) compared to the quartz-rich lighter areas (red arrows) (SG-278).

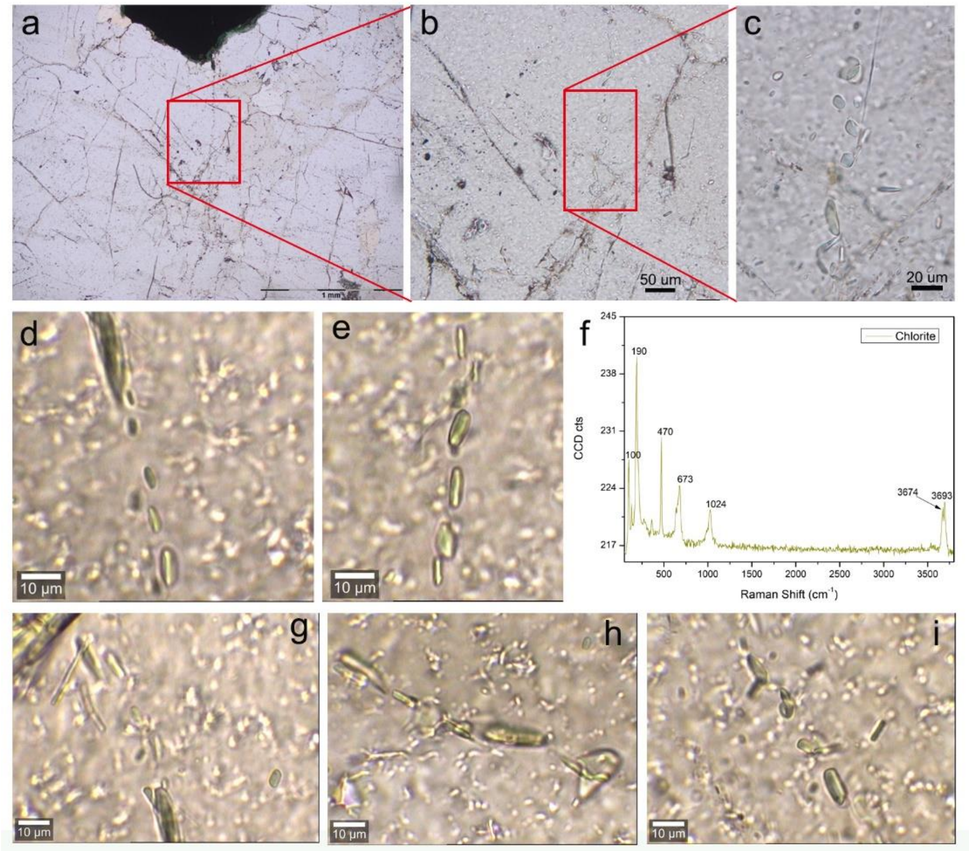

Supplementary Figure 2. Chlorites in the Saglek-Hebron banded iron formation sample *SG-274*. **a-e, g-i** Transmitted Light images showing that secondary, retrograde chlorite is commonly pale green and intrudes the quartz-rich band as linear array from the rims of grunerite-rich band. **f** Raman spectrum of chlorite.

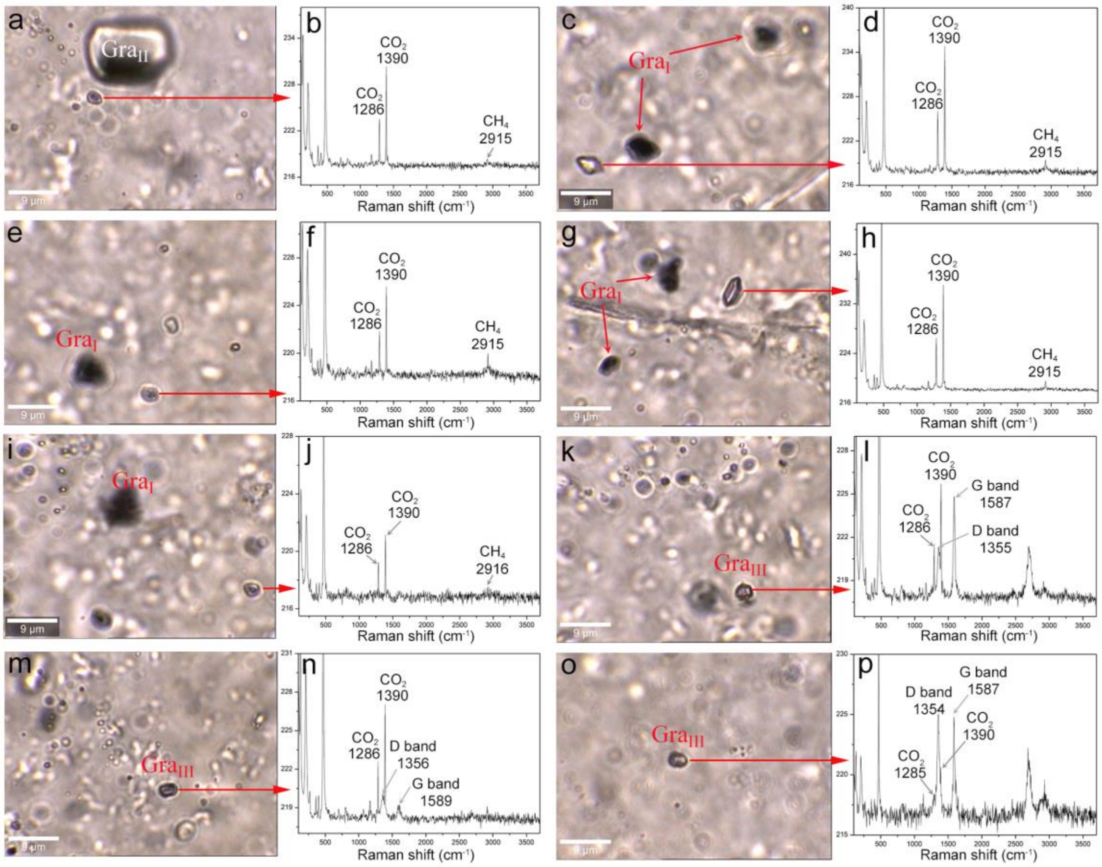

Supplementary Figure 3. Fluid inclusions in the Saglek-Hebron banded iron formation sample *SG-274*. **a** Transmitted Light (TL) image showing occurrences of calcite + graphite association (Gra_{II}) and fluid inclusions. **b** Raman spectrum of CO_2-CH_4 bearing fluid inclusion in a. **c**, **e**, **g**, **i** TL images showing occurrences of pure graphite (Gra_I) and fluid inclusions. **d**, **f**, **h**, **j** Raman spectra of CO_2-CH_4 bearing fluid inclusions in c, e, g, i, respectively. **k**, **m**, **o** TL images showing occurrences of graphite + fluid inclusions (Gra_{III}) and fluid inclusions. **l**, **n**, **p** Raman spectra of Gra_{III} in k, m, o, respectively.

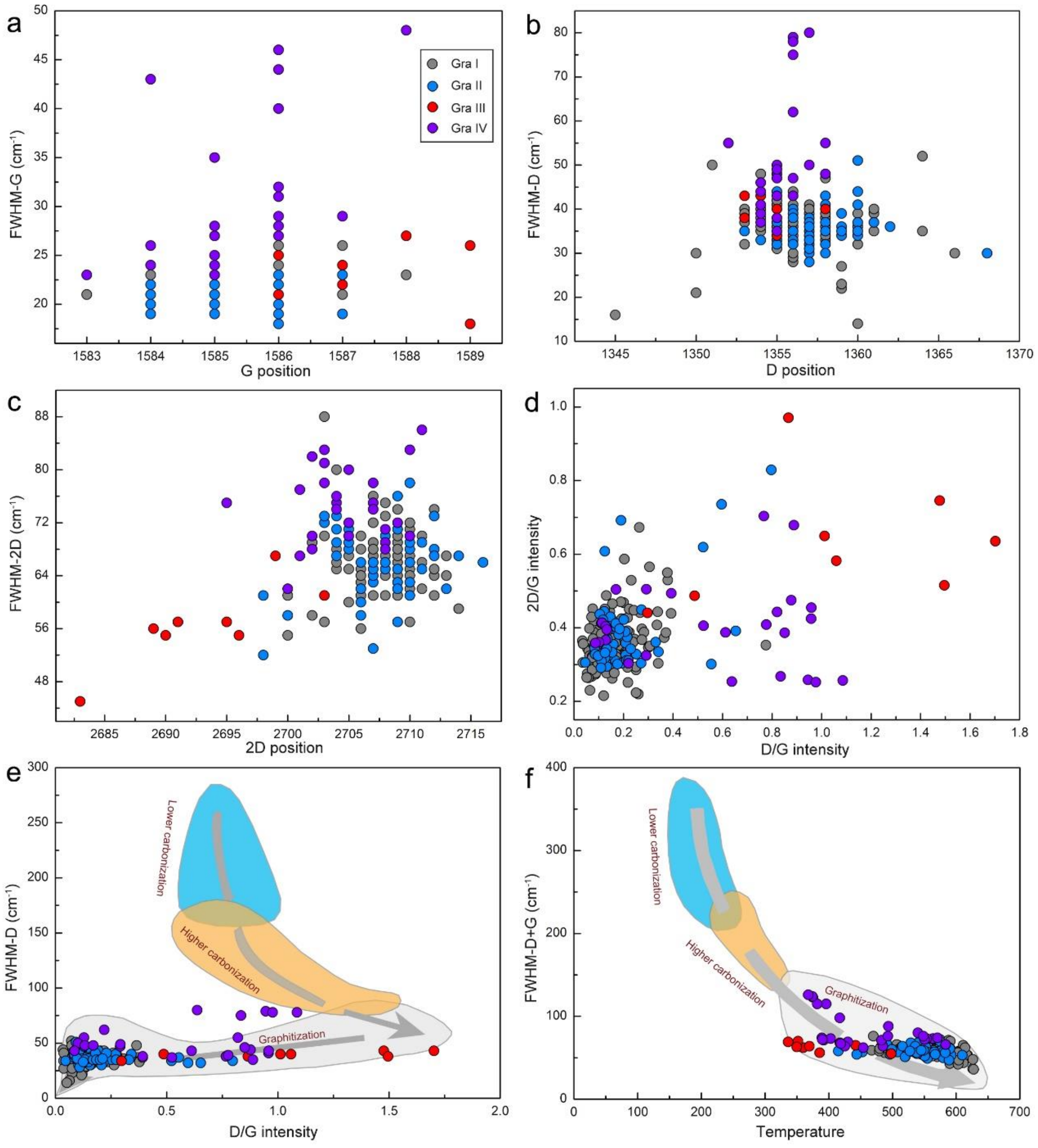

Supplementary Figure 4. Plots of Raman spectral characteristics for four types of graphite in the Saglek-Hebron rocks. a G-band FWHM (full width at half maximum) vs. G-band position. **b** D-band FWHM vs. D-band position. **c** 2D-band FWHM vs. 2D-band position. **d** 2D/D vs. D/G intensity ratio. **e** D-band FWHM vs. D/G intensity. **f** The sum of the FWHM at D and G pecks vs. the crystallization temperatures. Gray arrows point towards increasing maturation of organic matter in rocks. Data for the range of the three defined organic matter populations are from Nabhan and Canfield (2023)[14]. Note that most Gra_{III} and Gra_{IV} samples are significantly lower than the metamorphic temperature experienced by the host rocks during upper amphibolite facies metamorphism (500-640 °C), potentially indicating a secondary origin.

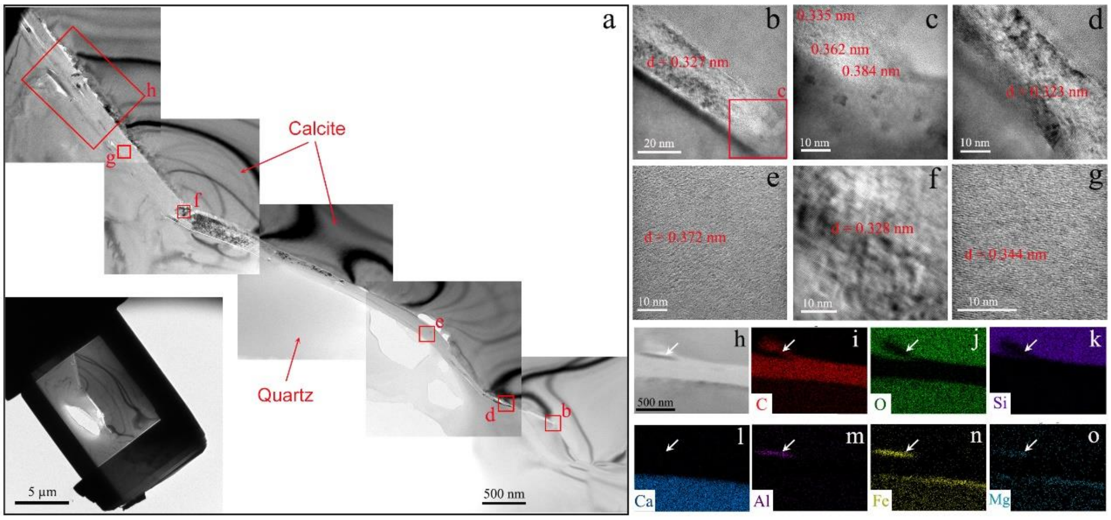

Supplementary Figure 5. Microscopic and compositional data of graphite in the Saglek-Hebron banded iron formation. a Bright-field TEM image mosaic showing graphite as an interlayer between quartz and calcite with a sharp contact boundary. **b-g** High-resolution TEM images showing the (002) interplanar spacings range from 3.23 Å to 3.84 Å in graphitic lattice fringes. **h-o** TEM image (h) and EDS mappings (i-o) for elemental distributions of carbon (red), oxygen (green), silicon (purple), calcium (blue), aluminum (pink), iron (yellow), and magnesium (cyan), revealing nano-chlorite (white arrow) embed into graphite.

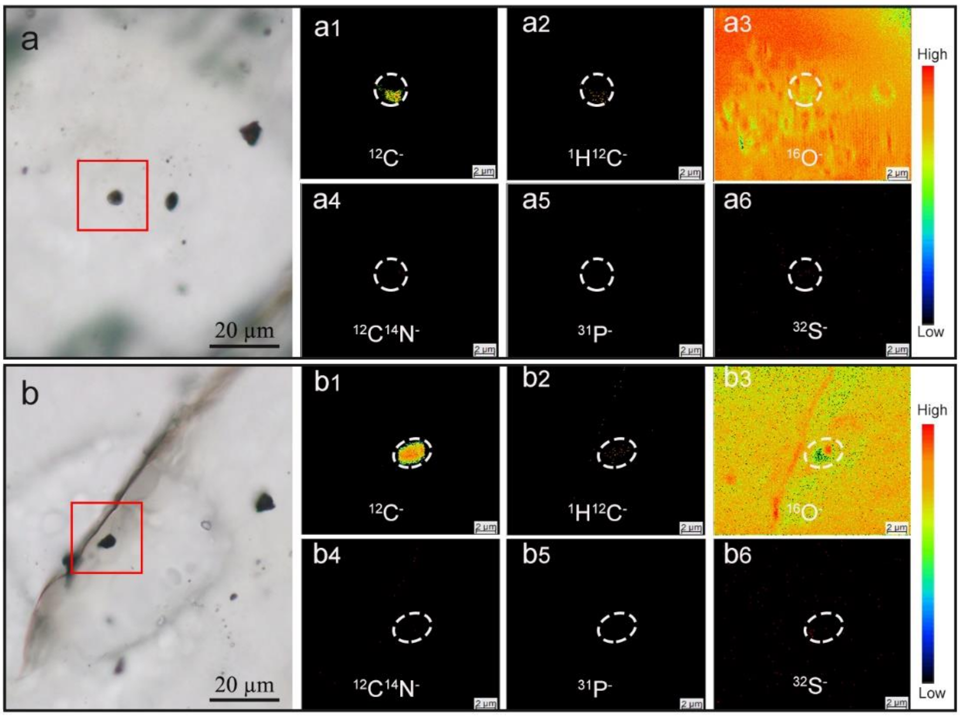

Supplementary Figure 6. Transmitted Light images and corresponding NanoSIMS elemental mappings of graphite in the Saglek-Hebron banded iron formation. a, b Transmitted Light images of graphite. **a1-a6** Elemental distributions of carbon (^{12}C), hydrogen (^{1}H), oxygen (^{16}O), nitrogen (^{14}N), phosphorus (^{31}P), and sulfur (^{32}S) of graphite in a. **b1-b6** Elemental distributions of carbon (^{12}C), hydrogen (^{1}H), oxygen (^{16}O), nitrogen (^{14}N), phosphorus (^{31}P), and sulfur (^{32}S) of graphite in b.

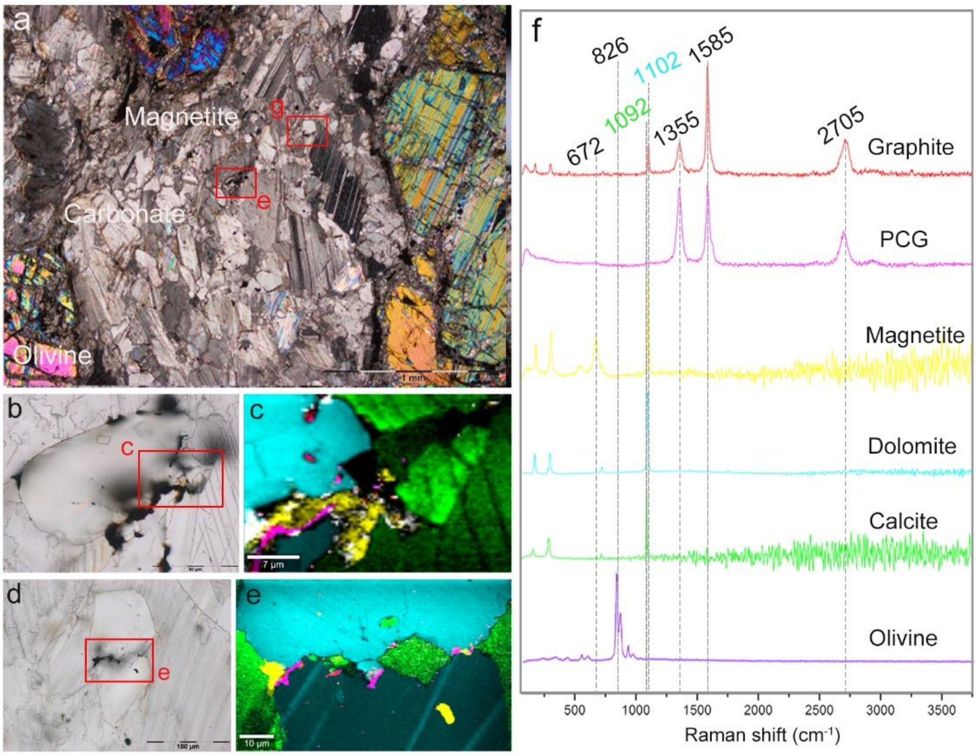

Supplementary Figure 7. Graphitic carbons and magnetite in the Saglek-Hebron marble sample *SG-275*. a Cross polar image showing occurrences of carbonate, magnetite and olivine with serpentine veins. **b-e** Transmitted Light (b and d) and Raman (c and e) images of opaque phases including micro- to nanometre size disseminations of graphite, PCG, and magnetite within and on the edges of carbonate grains. **f** Raman spectra for this figure. Raman map colours: red – graphite, pink – PCG, yellow – magnetite, turquoise – dolomite, green – calcite, and black – poor signal.

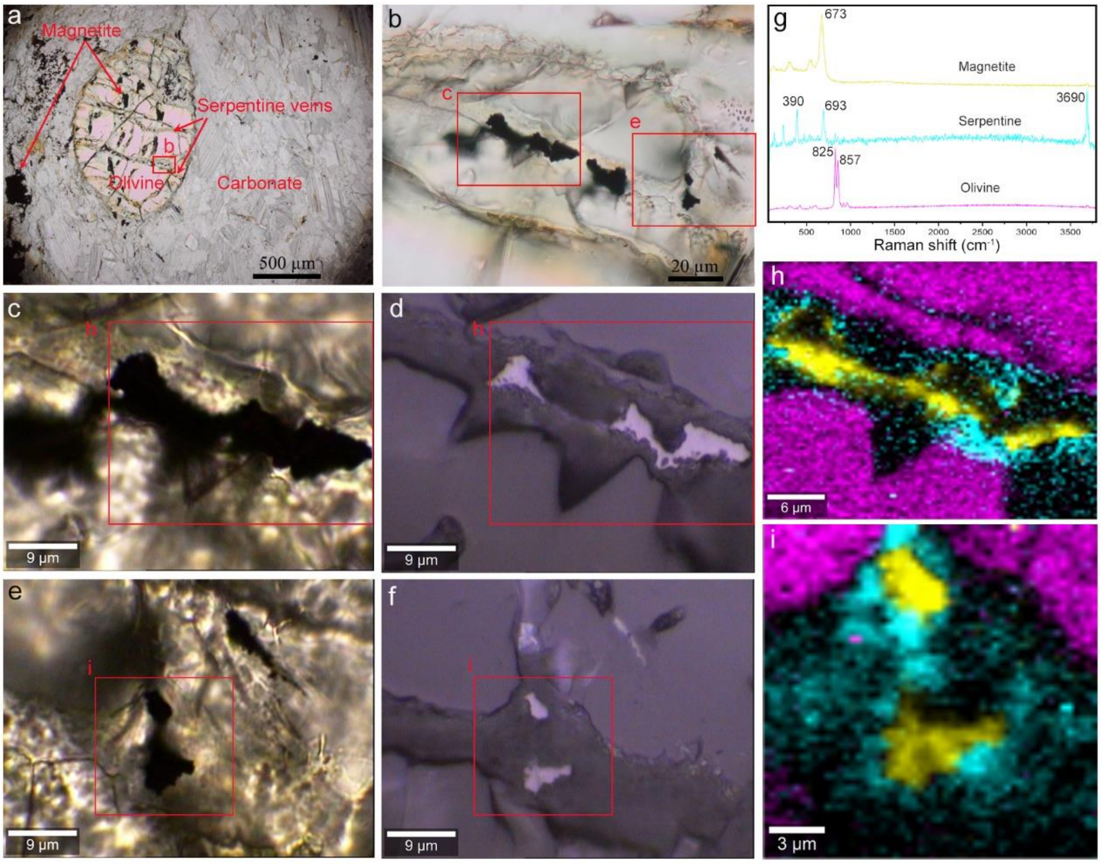

Supplementary Figure 8. Magnetite in serpentine veins in the Saglek-Hebron marble sample *SG-275*. **a** Plane Polarised Light (PPL) image showing occurrences of carbonate, magnetite, and olivine with serpentine veins. **b** PPL image showing typical magnetite in serpentine vein. **c-f** Enlarged views showing occurrences of typical magnetite under transmitted (c and e) and reflected light (d and f). **g** Raman spectra for this figure, corresponding to panels h-i in colour. **h-i** Raman images showing that magnetite in serpentine veins is not associated with graphite. Raman map colours: yellow – magnetite, pink – olivine, turquoise –serpentine, and black – poor signal.

Supplementary References

1. Bridgwater, D., Collerson, K.D., Hurst, R.W. & Jesseau, C.W. Field characters of the early precambrian rocks from Saglek, Coast of Labrador. *Geological Survey of Canada* **75 (1)**, 287–296 (1975).

2. Baadsgaard, H., Collerson, K.D. & Bridgwater, D. The Archean gneiss complex of northern Labrador. 1. Preliminary U-Th-Pb geochronology. *Can. J. Earth Sci.* 951–961 (1979).

3. Bridgwater, D. & Schiøtte, L. The Archean gneiss complex of northern Labrador. A review of current results, ideas and problems. *Bull. Geol. Soc. Den.* **39 (3–4)**, 153–166 (1991).

4. Kusiak, M.A. et al. Peak to post-peak thermal history of the Saglek Block of Labrador: A multiphase and multi-instrumental approach to geochronology. *Chem. Geol.* **484**, 210–223 (2018).

5. Sałacińska, A. et al. Complexity of the early Archean Uivak Gneiss: Insights from Tigigakyuk Inlet, Saglek Block, Labrador, Canada and possible correlations with south West Greenland. *Precambrian Res.* **315**, 103–119 (2018).

6. Wasilewski, B., O'Neil, J., Rizo, H., Paquette, J.-L. & Gannoun, A.-M. Over one billion years of Archean crust evolution revealed by zircon U-Pb and Hf isotopes from the Saglek-Hebron complex. *Precambrian Res.* **359**, 106092 (2021).

7. Wendt, J.I. & Collerson, K.D. Early Archean U/Pb fractionation and timing of late Archean high-grade metamorphism in the Saglek—Hebron segment of the North Atlantic Craton. *Precambrian Res.* **93**, 281–297 (1999).

8. Morino, P., Caro, G., Reisberg, L. & Schumacher, A. Chemical stratification in the post-magma ocean Earth inferred from coupled ^{146,147}Sm–^{142,143}Nd systematics in ultramafic rocks of the Saglek block (3.25–3.9 Ga; northern Labrador, Canada). *Earth Planet. Sci. Lett.* **463**, 136–150 (2017).

9. Morino, P., Caro, G. & Reisberg, L. Differentiation mechanisms of the early Hadean mantle: Insights from combined176Hf-142,143Nd signatures of Archean rocks from the Saglek Block. *Geochim. Cosmochim. Acta* **240**, 43–63 (2018).

10. Bridgwater, D. & Collerson, K.D. On the origin of Early Archean gneisses: a reply. Contrib. Miner. Petrol. 62, 179–191 (1977).

11. Shimojo, M. et al. Occurrence and geochronology of the Eoarchean, ~3.9 Ga, Iqaluk Gneiss in the Saglek Block, northern Labrador, Canada: Evidence for the oldest supracrustal rocks in the world. *Precambrian Res.* **278**, 218–243 (2016).

12. Whitehouse, M.J., Dunkley, D.J., Kusiak, M.A. & Wilde, S.A. On the true antiquity of Eoarchean chemofossils – assessing the claim for Earth's oldest biogenic graphite in the Saglek Block of Labrador. *Precambrian Res.* **323**, 70–81 (2019).

13. Schiøtte, L., Compston, W. & Bridgwater, D. U–Th–Pb ages of single zircons in Archaean supracrustals from Nain Province, Labrador, Canada. *Can. J. Earth Sci.* **26**, 2636–2644 (1989).

14. Nabhan, S. & Canfield, D.E. Estimating the upper limit of Proterozoic petrographic organic carbon recycling. *Precambrian Res.* **390**, 107034 (2023).
