# Supplementary material for: Abiotic synthesis of graphitic carbons in the Eoarchean Saglek-Hebron metasedimentary rocks
Source: Nat Commun. 2024 Jul 6;15:5679. doi: 10.1038/s41467-024-50134-1 (PMC11227522; doi:10.1038/s41467-024-50134-1)
Supplement: Supplementary file 3 — Description of Additional Supplementary Files [file 41467_2024_50134_MOESM3_ESM.pdf]

Description of Additional Supplementary Files

File Name: Supplementary Data 1
Description: Mineral compositions (wt%) for the Saglek-Hebron metasedimentary rocks determined by TIMA.

File Name: Supplementary Data 2
Description: Raman spectral characteristics of graphite in the Saglek-Hebron metasedimentary rocks. To estimate the maximum crystallization temperatures of graphite, we used the geothermometer calibrated for temperatures between 330°C and 641°C from Beyssac et al. (2002).

File Name: Supplementary Data 3
Description: Elemental compositions of various minerals in the Saglek-Hebron metasedimentary rocks analysed by TEM-EDS.
